# Cardiac rehabilitation after myocardial infarction: Insights into participation rates and predictors, sex differences and outcomes from a Dutch general hospital

**DOI:** 10.1016/j.ahjo.2026.100821

**Published:** 2026-06-25

**Authors:** Charlotte de Fouw, Ferry Hersbach, Esther Scheffer, Max W. Veninga, Clara E.E. van Ofwegen, M. Yldau van der Ende

**Affiliations:** a Division of Cardiology, Diakonessenhuis hospital, Bosboomstraat 1, 3582 KE, Utrecht, the Netherlands; b Department of Cardiology, Division Heart and Lungs, University Medical Center Utrecht, Utrecht University, Heidelberglaan 100, 3584 CX, Utrecht, the Netherlands

**Keywords:** Cardiac rehabilitation, Myocardial infarction, Secondary prevention, Sex characteristics, Risk factors, Predictors

## Abstract

**Background:**

Cardiac rehabilitation (CR) is an effective intervention to reduce major cardiac events following myocardial infarction (MI). Despite strong recommendations, participation in CR remains suboptimal. Especially among vulnerable patients like elderly. This study aims to determine contemporary CR participation rates, predictors of non-participation and sex-specific differences at a Dutch general hospital, while evaluating the impact of CR on major adverse cardiac events (MACE) and mortality. Method: A single-center retrospective cohort study included patients admitted to Diakonessenhuis (Utrecht, Netherlands) from December 2019–June 2022. Information regarding CR participation, patient characteristics (e.g. age, sex, medical history), psychological assessments and the incidence of MACE during follow-up were collected from patient files.

**Result:**

Of 271 included patients, 63.5% participated in CR. Predictors of CR participation included younger age and undergoing coronary artery bypass grafting (CABG). Female patients were older (68.8 vs 64.8 years, *p* = 0.013), had worse scores on psychological questionnaires during CR and showed lower CR completion rates (71.8% vs 82.0%, *p* = 0.025) compared to men. CR participation was associated with lower all-cause mortality (HR 0.16, 95% CI 0.02–0.78, *p* = 0.023) during a median follow-up time of 4.1 years.

**Conclusion:**

CR participation after MI remains suboptimal. Younger age and CABG are associated with higher participation rates. Female patients show lower CR completion rates and perform worse psychologically. Tailored interventions should address compounded barriers such as older age and possible sex-specific psychosocial burdens.

## Highlights

• The participation rate in cardiac rehabilitation is around 65%
• Younger age and male sex are associated with higher participation rates to cardiac rehabilitation
• Coronary artery bypass grafting as primary intervention predicts participation
• Men have a higher cardiac rehabilitation completion rate than do women
• Participation in cardiac rehabilitation is associated with lower rates of MACE

## Graphical abstract

**Figure:**
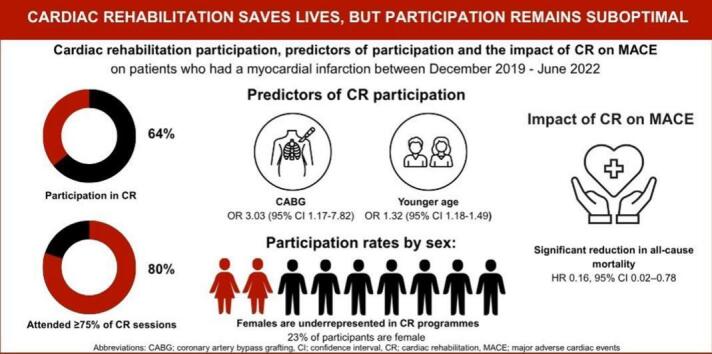

## 1 Introduction

Cardiovascular disease (CVD) is a leading global health concern, responsible for over 2 million deaths annually in Europe. [1] Nearly half of these deaths are due to ischemic heart disease (IHD), making it the primary contributor to CVD mortality. [2] Since the 1980s, advancements in clinical care—such as defibrillation, revascularization techniques, and novel medications [3]—have led to a 30–70% reduction in CVD [1] and IHD [4] mortality rates. These improvements have increased survival and the number of patients living with chronic cardiovascular conditions, resulting in a higher demand for long-term care and placing a growing burden on healthcare systems.

Given this higher demand, secondary prevention is increasingly important. Cardiac rehabilitation (CR) is a cornerstone of secondary prevention following IHD^.^ It is a comprehensive, patient-centered program involving physical training, health education, cardiovascular risk management, and psychological support. [5] Participation in CR has been shown to reduce mortality and hospital readmissions, lower the risk of recurrent cardiac events, and significantly improve quality of life. [6] As such, CR is strongly recommended in international guidelines (Class IA). [7]

Despite its benefits, CR utilization remains suboptimal, with participation influenced by patient-level factors such as age, sex, socioeconomic status (SES), travel distance, revascularization type, history of CVD, and marital status. [8], [9] Studies have consistently shown that women are less likely to be referred to, enroll in, and complete CR, despite having a greater need due to higher rates of comorbidities, lower functional capacity, and greater psychosocial burdens, including depression and low SES. [10], [11] This treatment–risk paradox has been acknowledged for over a decade, prompting sex-specific guideline recommendations, though the effectiveness of these efforts remains unclear. [12]

This study aims to determine current CR participation rates, identify their predictors, and evaluate their impact on major adverse cardiovascular events (MACE) and mortality at Diakonessenhuis, a large general hospital in Utrecht, the Netherlands. It also explores sex-specific barriers to CR completion. This study thus provides contemporary hospital-specific insights in the context of the Dutch publicly funded healthcare system, including less well-studied predictors such as marital and retirement status.

## 2 Methods

### 2.1 Study design and data collection

We conducted a single-center retrospective observational study using a registry of patients admitted for acute coronary syndrome (ACS) at Diakonessenhuis Utrecht between December 2019 and June 2022. Adults (≥18 years) diagnosed with ST-elevation myocardial infarction (STEMI) or non-ST elevation myocardial infarction (NSTEMI) were included. Patients were excluded if CR program data were incomplete or follow-up occurred at another hospital. Follow-up spanned from admission to April 1, 2025.

This retrospective study used patient data extracted from electronic medical records and included demographics (e.g. age, sex, marital status, occupation, primary revascularization therapy) and medical information (e.g. comorbidities, physical activity, blood pressure). In this study, sex referred to biological attributes associated with physical and physiological features (male/female based on assigned sex at birth); gender was not assessed, as this study focused on clinical outcomes rather than socially constructed roles.

All data were de-identified and no patient interventions or prospective data collection occurred. Data sensitive to re-identification, such as postal codes, were aggregated to ensure privacy protection.

### 2.2 Cardiac rehabilitation program at Diakonessenhuis Utrecht

All patients admitted with IHD were provided with information on CR and referred to a CR center. Upon arrival, they completed lifestyle (e.g. smoking, physical activity, alcohol use) and psychological questionnaires including:

• MacNew Heart Disease QoL Questionnaire: Assessed physical, emotional, and social functioning post-MI. Scores range from 27 to 189; higher scores reflect better quality of life. The minimal important difference is 13 points. [13]
• Hospital Anxiety and Depression Scale (HADS): Screened for anxiety and depression. Scores range from 0 to 21. Scores of 8 or higher were used to classify patients with symptoms of depression or anxiety.
• Patient Health Questionnaires (PHQ-2 and PHQ-9): Evaluated depressive symptoms. PHQ-9 scores ≥5 indicate depression (range 0–27), PHQ-2 scores ≥3 indicate that a major depressive disorder is likely (ranges 0–6).

All patients also completed an ergometric test to determine their metabolic equivalent of task (METs).

Based on individual goals and assessments, patients were assigned to one of six functional capacity fit groups (1 = highest, 6 = lowest) by a multidisciplinary team (cardiologist, nurse practitioner, physiotherapist, psychologist). A capacity fit group is an estimation of an individual's functional capacity for exercise and exertional endurance.

The CR program included three modules:

• Lifestyle module: Five two-hour sessions led by a psychologist and social worker focused on stress management and boundary setting.
• Information module: Four self-study presentations by a nutritionist, psychologist, and social worker on the impact of myocardial infarction and future implications for patients' health.
• Fitness module: Eleven to sixteen one-hour physiotherapy sessions, with functional capacity assessed before and after the program.

### 2.3 Definitions

Cardiovascular risk factors were defined as:

• **Hypertension**: systolic >130 mmHg and/or diastolic >80 mmHg [14].
• **Heavy alcohol use**: >6 drinks per week [15].
• **Physical activity**: scored as 1 (none), 2 (1–2 times/week), or 3 (≥3 times/week).

MACE included any of the following: acute myocardial infarction, unstable angina, ventricular arrhythmias, stroke, revascularization, or all-cause mortality. [16]

SES was assessed using the SES-WOA score from Statistics Netherlands (CBS), based on the participant's home postal code. This scoring system contains information on the average wealth, education level, and employment status of inhabitants of that postal code. [17] Scores were grouped into low, moderate, and high SES. Country of birth and language barriers during treatment were also recorded.

### 2.4 Statistical analysis

Quantitative variables were reported as mean with standard deviation (SD) if they were normally distributed. Non-normally distributed variables were reported using median with interquartile ranges (IQR). Categorical and dichotomous variables were expressed as percentages.

The Chi-squared test was used to compare categorical variables. For continuous variables, either an independent samples *t*-test (if the data was normally distributed), or the Mann–Whitney *U* test (if the data was not normally distributed) was used.

To identify potential predictors of participation to rehabilitation, univariate logistic regression analyses were first performed. Variables with a *p*-value below 0.10 in the univariate analysis were included in a multivariable logistic regression model. A backward stepwise method was applied, with variables being removed if *p* ≥ 0.10 and considered statistically significant if *p* < 0.05. Additionally, a cox proportional hazards regression analysis was performed to determine the association between CR participation and MACE and all-cause mortality. We examined the interaction of the sex variable by adding product terms of sex and CR participation to the cox regression models.

All statistical analyses were performed using StataNow version 18.5. Throughout, a two sided *p*-value of less than 0.05 was considered statistically significant.

## 3 Results

### 3.1 Study population

**Fig. 1:**
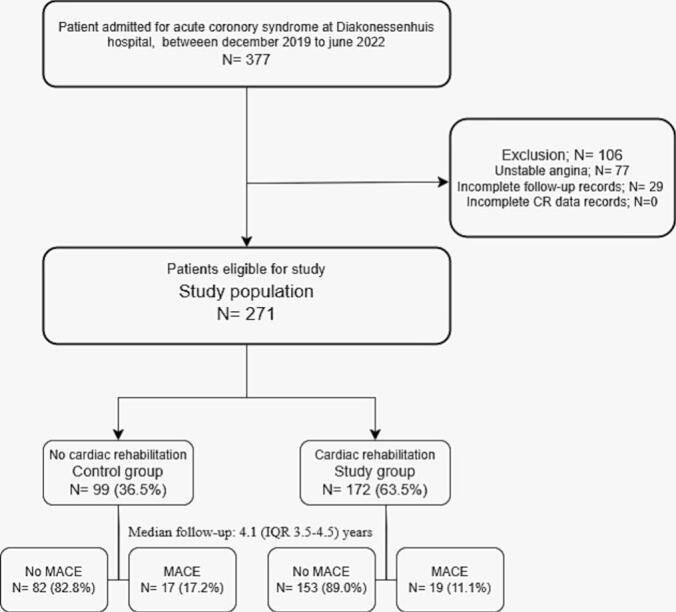
Study design flowchart.

Between December 2019 and June 2022, 377 patients were admitted to the cardiology department of Diakonessenhuis Utrecht with ACS. Of these, 106 were excluded: 77 due to unstable angina pectoris and 29 due to incomplete follow-up data (Fig. 1).

### 3.2 Patient baseline characteristics

The baseline characteristics of the study population are presented in Table 1. Of the 271 patients included in this study, 172 (63.5%) participated in CR after admission. Among participants, 79.7% completed the program with >75% attendance to the program sessions, aligning with prior Dutch studies. [20] At baseline, certain individual factors were present more often in those who did not participate in CR, and vice versa. Compared to non-participants, the CR participation group featured less women (22.7% vs 35.5%, *p* < 0.024), were younger (63.5 (SD 10.7) years vs. 70.1 (SD 12.5) years, *p* < 0.001), and more often had received a coronary artery bypass grafting (CABG) as revascularization intervention. Additionally, CR-participants were more likely to be married (76.2% vs. 47.5%, *p* < 0.001) and have current employment (50% vs. 21.2%, *p* < 0.001). Conversely, non-participants had lower levels of alcohol consumption (47.5% vs 63.4%, *p* = 0.040) and more frequently had a history of thoracic angina (20.2% vs 11.1, *p* = 0.039) or a history of CABG (0.0% vs 4.0%, *p* = 0.008).

**Table 1:** Baseline characteristics, divided by CR participation.

Variables	Total	CR participation	No-CR participation	P-value
	*N* = 271	*N* = 172 (63.5%)	*N* = 99 (36.5%)	
Demographic factors, N (%)				
Female sex	74 (27.3)	39 (22.7)	35 (35.5)	**0.024**
Age (mean (SD)), years	65.93 (11.8)	63.5 (10.7)	70.1 (12.5)	**<0.001**
Unmarried^a^	42 (15.5)	30 (17.4)	12 (12.1)	0.244
Married^a^	178 (65.7)	131 (76.2)	47 (47.5)	**<0.001**
Widow^a^	21 (7.8)	8 (4.7)	13 (13.1)	**0.012**
Foreign born	41 (15.1)	28 (16.4)	13 (13.1)	0.495
Language barrier	19 (7.0)	11 (6.4)	8 (8.1)	0.585

Cardiovascular risk factors, N (%)				
Hypertension	105 (38.9)	62 (36.3)	43 (43.4)	0.244
Diabetes Mellitus	39 (14.4)	20 (11.6)	19 (19.2)	0.088
BMI, median (IQR)	26 (24–29)	26.4 (24.3–29.5)	25.8 (23–29)	**0.055**
Active smoking	74 (27.3)	50 (29.1)	24 (24.2)	0.390
Alcohol use	156 (57.6)	109 (63.4)	47 (47.5)	**0.040**

Type of event, N (%)				
NSTEMI	155 (57.2)	98 (57.0)	57 (57.6)	0.924
STEMI	116 (42.8)	74 (43.0)	42 (42.4)	0.924

Cardiac intervention, N (%)				
PCI	218 (80.4)	140 (81.9)	78 (78.8)	0.536
CABG	31 (11.4)	25 (14.7)	6 (6.1)	0.034
None	31 (11.4)	15 (8.7)	16 (16.2)	0.064

Measurements, mean (SD)				
TC, mmol/L	4.8 (1.2)	4.79 (1.1)	4.97 (1.3)	0.274
SBP, mmHg (median - IQR)	142 (127–160)	141.5 (125–159)	143 (128–164)	0.145
DBP, mmHg	86.8 (15.6)	87.7 (15.7)	85.1 (15.5)	0.180

Previous CVD history, N (%)				
CABG	4 (1.5)	0 (0)	4 (4.0)	**0.008**
PCI	32 (11.8)	16 (9.3)	16 (16.2)	0.092
ACS	37 (13.7)	17 (9.9)	20 (20.2)	0.017
Angina pectoris	39 (14.4)	19 (11.1)	20 (20.2)	0.039
PAD	14 (5.2)	10 (5.8)	4 (4.0)	0.525
Stroke	6 (2.2)	4 (2.3)	2 (2.0)	0.869
CKD	11 (4.1)	6 (3.5)	5 (5.1)	0.530

Logistical, median (25th and 75th percentiles)				
Distance to CR, km	6.4 (3.3–10.1)	5.8 (3.1–9.8)	7 (3.7–12.9)	0.161
Driving distance, km	9.5 (4.9–14.2)	9.32 (4.6–12.8)	9.8 (5.2–16.8)	0.153
Driving duration, min	12 (8.1–16.2)	11.8 (7.9–15)	12.1 (8.8–19.7)	0.164

Social economic status, N (%)				
Low	89 (32.8)	60 (34.9)	29 (29.3)	0.345
Moderate	90 (33.2)	59 (34.3)	31 (31.3)	0.615
High	92 (34.0)	53 (30.8)	39 (39.4)	0.151

Occupation, N (%)				
Employed^b^	107 (39.5)	86 (50.0)	21 (21.2)	**<0.001**
Un-employed^b^	43 (15.9)	29 (17.0)	14 (14.1)	0.555
Retired^b^	89 (32.8)	49 (28.5)	40 (40.4)	**0.044**

Abbreviations: SD, standard deviation; BMI, body mass index; PCI, percutaneous coronary intervention; SBP, systolic blood pressure; DBP, diastolic blood pressure; CABG, coronary artery bypass graft surgery; TC, total cholesterol; IQR, interquartile range; ACS, acute coronary syndrome; PAD, peripheral artery disease; CKD, chronic kidney disease.

P-values <0.05 were considered to be statistically significant.

a Data missing for 30 patients.

b Data missing for 32 patients.

### 3.3 Predictors of CR non-participation

In the univariate logistic regression analysis on CR participation, female sex (OR 1.85 (95% CI 1.04–3.23, *p* = 0.025), widowed (OR 4.55 (95% CI 1.75–11.11), *p* = 0.002), retired (OR 3.33 (95% CI 1.79–6.25), *p* < 0.001), older age (OR 1.30 per 5 years (95% CI 1.15–1.45), *p* < 0.001) or having a history of previous ACS (OR 2.33 (95% CI 1.15–4.76), *p* = 0.019) or previous thoracic angina (OR 2.04 (95% CI 1.03–4.00), *p* = 0.041) were associated with higher odds of non-participation in CR (Table 2).

**Table 2:** Univariate and multiple logistic regression analyse for predictors of CR non-participation.

	Univariate logistic regression			Multiple logistic regression		
	P-value	Odds Ratio	95% CI	P-Value	Odds Ratio	95% CI
Demographic factors						
Female	**0.025**	1.85	1.04–3.23			
Age, per 5 years	**<0.001**	1.30	1.15–1.45	**<0.001**	1.32	1.18–1.49
Married (ref)						
Unmarried	0.776					
Widow	**0.002**	4.55	1.75–11.11			

Cardiovascular risk factors						
Hypertension	0.244					
Diabetes Mellitus	**0.090**	1.82	0.91–3.57			
BMI	0.113					
Active smoking	0.391					
Stopped smoking after event	0.133					
Alcohol use	**0.025**	0.56	0.34–0.93			

Type of event						
NSTEMI/STEMI	0.924					

Cardiac revascularisation intervention						
PCI	0.536					
CABG	**0.040**	0.38	0.15–0.96	**0.022**	0.33	0.13–0.85
None	**0.068**	2.00	0.95–4.35			

Previous CVD history						
CABG	n.c.⁎					
PCI	**0.096**	1.89	0.89–4.00			
ACS		2.33	1.15–4.76			
Angina pectoris	**0.041**	2.04	1.03–4.00			
PAD	0.528					
Stroke	0.869					

Social economic status						
Low (ref)						
Moderate	0.582					
High	0.407					

Occupation						
Employed (ref)						
Unemployed	0.094	1.96	0.89–4.35			
Retired	**<0.001**	3.33	1.79–6.25			

Abbreviations: ref., reference; BMI, body mass index; NSTEMI, non-ST elevated myocardial infarction; STEMI, ST elevated myocardial infarction; CABG, coronary artery bypass graft surgery; PCI, percutaneous coronary intervention; PAD, peripheral artery disease.

A backward stepwise method was applied, with variables being removed if *p* ≥ 0.10 and considered statistically significant if *p* < 0.05.

⁎ P-value could not be calculated due to a zero-cell count.

Factors that were associated with a higher participation rate in CR were frequent alcohol use (OR 1.79 (95% CI 1.08–2.97), *p* = 0.025) and receiving a CABG as primary intervention (OR 2.64 (95% CI 1.04–6.69), *p* = 0.040).

In the multiple logistic regression analysis, only younger age (OR 0.76 (95% CI 0.67–0.85), *p* < 0.001) and receiving a CABG as primary intervention (OR 3.03 (95% CI 1.17–7.82), *p* = 0.022) were associated with CR participation (Table 2).

### 3.4 Sex-based differences in cardiac rehabilitation variables

Multiple CR related variables were examined with sex-specific comparisons and are presented in Table 3. Patients attended 8.5 (SD 2.7) sessions on average. Male patients were more likely to complete the program (82% vs. 71.8%, *p* = 0.025), defined as attending at least 75% of all planned sessions. Women had a lower baseline fitness based on METs. (6.0 (SD 1.9) vs. 4.6 (SD 1.7), *p* < 0.001) and were more frequently divided into lower fit groups. Female patients reported worse scores on psychological questionnaires, including the HADS scale (*p* = 0.141 and *p* < 0.005) and PHQ-2 (*p* < 0.005) and 9 (*p* = 0.01), but scored higher on quality of life (*p* = 0.026).

**Table 3:** Baseline CR characteristics, divided by sex.

Variables	CR rehabilitation participants			
	Total (N = 172)	Male (*N* = 133)	Female (*N* = 39)	*P*-value
Participation rates, mean (SD)				
Average session attendance	8.5 (2.9)	8.5 (2.7)	8.2 (3.6)	0.508
CR completion, >75% attendance (N (%))	137 (79.7)	109 (82.0)	28 (71.8)	0.025
Average number of CR sessions	9.7 (3.1)	9.76 (2.9)	9.28 (3.8)	0.392

Psychological questionnaires, mean (SD)				
QoL total score	126.9 (23.2)	129.3 (23.3)	118.9 (21.5)	0.026
HADS depression score	3.5 (3.9)	3.3 (3.7)	4.4 (4.4)	0.141
HADS anxiety score	4.8 (3.9)	4.3 (3.7)	6.4 (4.5)	0.0048
PHQ-2 score	1.3 (1.5)	0.9 (1.2)	1.7 (2.0)	0.0025
PHQ-9 score	4.2 (4.4)	3.7 (4.0)	6.0 (5.3)	0.010

Physical measurements, N (%)				
Level of physical activity at baseline				
Activity score 1^a^	35 (20.4)	29 (21.8)	6 (15.4)	0.381
Activity score 2^a^	46 (26.7)	36 (27.1)	10 (25.6)	0.860
Activity score 3^a^	42 (24.4)	33 (24.9)	9 (23.1)	0.824
Metabolic equivalent of task (mean ± SD)	5.6 (1.9)	6.0 (1.9)	4.6 (1.7)	<0.001
FIT group CR (mean ± SD)	3.0 (1.8)	2.6 (1.6)	4.1 (1.95)	<0.001

Abbreviations: SD, standard deviation; CR, cardiac rehabilitation; QoL, quality of life; HADS, hospital anxiety depression scale; PHQ, patient health questionnaire.

a Data missing for 42 patients.

### 3.5 Clinical outcomes

During a median follow-up of 4.1 (IQR 3.5–4.5) years, a total of 36 (13.3%) patients had been diagnosed with MACE (supplementary Table 1). A total of 19 (11.1%) of these were in the CR participation group vs. 17 (17.2%) in the non-CR participation group (*p* *=* *0.153)*. Cox proportional hazards regression analysis showed no association between CR participation and MACE (*p* = 0.182). There were no significant interactions between sex and MACE between individuals with CR participation or without participation. All-cause mortality occurred in 2 patients (1.2%) in the CR group vs 7 patients (7.1%) in the non-CR group (*p* = 0.009*).* Additionally, in cox regression analysis on all-cause mortality, CR participation was associated with lower all-cause mortality (HR of 0.16, 95% CI 0.023–0.778; *p* *=* *0.023*).

## 4 Discussion

### 4.1 Novelty of findings

This study provided current data regarding CR participation rates, predictors of non-participation and explored sex-specific differences. We found that participation rates continue to be suboptimal. Younger age and CABG as the primary revascularization intervention were predictors of participation in CR. Female CR participants scored lower on psychological and fitness tests. Lastly, participation in CR was associated with reduced mortality.

### 4.2 Predictors of CR participation

The CR participation and completion rates in this study (Table 2) align with previous studies. [18], [19], [20]. Given the identified predictors for non-participation—particularly older age —targeted strategies are needed to improve participation among vulnerable groups. Seminars for medical staff on CR benefits, combined with tailored oral or written patient education, could address knowledge gaps, as low-level interventions have nearly doubled participation rates in prior research. [21], [22] Additionally, cardiac telerehabilitation offers a promising alternative for older women and retired patients facing transport or scheduling barriers, providing equivalent efficacy to center-based programs. [22]

Each 5-year increase in age reduced the odds of CR participation. Older age is a known predictor and likely reflects barriers such as comorbidities, transportation challenges, exercise misconceptions and lower expectations of benefit. [23], [24], [25] Conversely, CABG as the primary revascularization method was a strong predictor of participation in the CR program. CABG patients may show greater motivation due to increased physical and emotional recovery needs. In the multivariate analysis, female sex was not an independent predictor after adjustment. This could reflect possible confounding due to later CVD onset, greater age-related barriers and lower CABG rates (Supplementary Table 2) [26], [27].

### 4.3 Sex differences in CR participants

Women who enrolled in CR exhibited lower completion rates than men and lower MET scores, indicating reduced maximal exercise capacity. Consequently, they were more frequently placed in lower fitness categories.

Female patients were also more affected by psychosocial factors, such as being unmarried (and lacking spousal support) and experienced higher levels of anxiety or depression. These findings are consistent with previous research showing that women participating in CR experience a higher psychological symptom burden and lower physical fitness levels than men, which may contribute to barriers in participation, completion and adherence [28] These disparities underscore the need for targeted psychological support and physical training for women in CR programs, specifically addressing the unique barriers women face.

### 4.4 The effect of CR on MACE and mortality

This study demonstrates that CR participation was associated with reduced all-cause mortality, consistent with existing literature. However, the analysis is likely underpowered due to the low event rate during follow-up. Although the link between CR and MACE was not statistically significant, a numerical trend toward fewer MACE events in CR participants was observed—supporting earlier studies showing a 20–30% reduction in MACE and mortality with CR. [17], [18]

### 4.5 Strengths and limitations

Limitations include potential residual confounding, as participation in cardiac rehabilitation was not determined by randomized allocation. Additionally, unmeasured factors such as left ventricular ejection fraction, concomitant use of cardiovascular (risk-reduction) medications, blood lipid data, a detailed record of administered exercise dose, health literacy and social support, which may have influenced (and potentially reduced) CR participation and outcomes. Second, the low event rate of all-cause mortality likely limited statistical power of corresponding analyses. Third, most inclusions occurred during the COVID-19 pandemic, which has undoubtedly impacted CR delivery (with non-essential inpatient care being reduced as much as possible) and participation, and therefore also the potential impact that CR could have had on patients. This has the potential to limit the generalizability of study findings to non-pandemic settings, which might show different results on CR participation factors, adherence and outcomes. Finally, findings derived from this single-center Dutch general hospital population may not be fully generalizable to other healthcare systems or places with different referral patterns.

Future research on the topic of cardiac rehabilitation could benefit from more comprehensive data collection of participants (e.g. graded-tests pre- and post CR, changes in individual factors such as blood pressure, smoking habits, blood lipid data, exercise regimens) to provide a more comprehensive overview on factors that might influence CR-participation and subsequent outcomes in order to improve the effectivity of CR-programs throughout.

## CRediT authorship contribution statement

**Charlotte de Fouw:** Writing – original draft, Methodology, Investigation, Formal analysis, Data curation. **Ferry Hersbach:** Writing – original draft, Resources, Project administration, Investigation. **Esther Scheffer:** Writing – original draft, Methodology, Investigation, Data curation, Conceptualization. **Max W. Veninga:** Writing – original draft, Project administration, Data curation. **Clara E.E. van Ofwegen:** Validation, Supervision, Methodology. **M. Yldau van der Ende:** Writing – review & editing, Supervision, Project administration.

## Ethical statement

Hereby, I (M.Y. van der Ende) assure that for the manuscript “Cardiac Rehabilitation after Myocardial Infarction: Insights into Participation Rates, Predictors and Sex Differences” the following is fulfilled:

• the work described has not been published previously except in the form of a preprint, an abstract, a published lecture, academic thesis or registered report.
• the article is not under consideration for publication elsewhere.
• the article's publication is approved by all authors and tacitly or explicitly by the responsible authorities where the work was carried out.
• if accepted, the article will not be published elsewhere in the same form, in English or in any other language, including electronically, without the written consent of the copyright-holder.

## Funding

This research did not receive any specific grant from funding agencies in the public, commercial, or not-for-profit sectors.

## Declaration of competing interest

C. de Fouw, F. Hersbach, E Scheffer, C.E.E. van Ofwegen, M.W. Veninga and M.Y. van der Ende declare that they have no competing interests or relationships that could be construed as a conflict of interest.

## Data Availability

Raw research data cannot be shared publicly due to privacy concerns associated with sensitive patient information from electronic medical records, in compliance with Dutch healthcare regulations and WMO exemption requirements. Aggregated results are fully reported in the tables.
