# Supplementary material for: Cardiac rehabilitation after myocardial infarction: Insights into participation rates and predictors, sex differences and outcomes from a Dutch general hospital
Source: Am Heart J Plus. 2026 Jun 25;68:100821. doi: 10.1016/j.ahjo.2026.100821 (PMC13330696; doi:10.1016/j.ahjo.2026.100821)
Supplement: Supplementary file 1 — Supplementary material [file mmc1.docx]

### *Supplementary materials*

**Supplementary Tab. 1** MACE for the total cohort and comparison between sex

|  | **CR participation** | | | | **Non-CR participation** | | | |
| --- | --- | --- | --- | --- | --- | --- | --- | --- |
| Variables, N (%) | Total (N=172) | Male (N=133) | Female (N= 39) | P-value | Total (N= 99) | Male (N= 64) | Female (N= 35) | P-value |
| *Any MACE* | 19 (11.1) | 13 (9.8) | 6 (15.4) | 0.326 | 17 (17.2) | 10 (15.6) | 7 (20.0) | 0.581 |
| *AMI* | 3 (1.7) | 2 (1.5) | 1 (2.6) | 0.656 | 5 (5.1) | 3 (4.7) | 2 (5.7) | 0.824 |
| *Ventricular arrhythmias* | 1 (0.6) | 0 (0.0) | 1 (2.7) | 0.064 | 0 (0.0) | 0 (0.0) | 0 (0.0) |  |
| *Revascularization* | 6 (3.5) | 3 (2.3) | 3 (7.7) | 0.104 | 7 (7.1) | 5 (7.8) | 2 (5.7) | 0.697 |
| *Unstable angina* | 5 (2.9) | 3 (2.3) | 2 (5.1) | 0.348 | 4 (4.0) | 2 (3.1) | 2 (5.7) | 0.532 |
| *Stroke* | 5 (2.9) | 4 (3.0) | 1 (2.6) | 0.885 | 1 (1.0) | 1 (1.6) | 0 (0.0) | 0.457 |
| *All‐cause mortality* | 2 (1.2) | 2 (1.5) | 0 (0.0) | 0.441 | 7 (7.1) | 4 (6.3) | 3 (8.6) | 0.667 |

Abbreviations: CR, cardiac rehabilitation; MACE, major adverse cardiac events; AMI, acute myocardial infarction.

**Supplementary Tab. 2** Baseline characteristics by sex

|  | Total | Female | | Male | P-value |
| --- | --- | --- | --- | --- | --- |
| Variables | N=271 | N= 74 (27.3%) | | N= 197 (72.7%) |  |
| ***Demographic factors, N (%)*** | | |  | | |
| *CR participation* | 172 (63.5) | 39 (52.7) | | 133 (67.5) | 0.024 |
| *Age (mean (SD))* | 65.9 (11.8) | 68.8 (12.4) | | 64.8 (11.4) | 0.013 |
| *Married** | 178 (65.7) | 30 (40.5) | | 148 (75.1) | <0.001 |
| *Unmarried** | 42 (15.5) | 16 (21.6) | | 26 (13.2) | 0.088 |
| *Widow** | 21 (7.78) | 14 (18.9) | | 7 (3.6) | <0.001 |
| *Foreign born* | 42 (15.6) | 11 (14.9) | | 31 (15.8) | 0.847 |
| *Language barrier* | 20 (7.4) | 5 (6.8) | | 15 (7.6) | 0.810 |
| ***Occupation, N (%)*** | | | | | |
| *Employed*** | 107 (39.5) | 22 (29.7) | | 85 (43.2) | 0.044 |
| *Un-employed*** | 43 (15.89) | 16 (21.6) | | 27 (13.7) | 0.112 |
| *Retired*** | 89 (32.8) | 21 (28.4) | | 68 (34.5) | 0.338 |
| ***Cardiovascular risk factors, N (%)*** | | | | | |
| *Hypertension* | 105 (38.9) | 34 (46.0) | | 71 (36.2) | 0.144 |
| *Diabetes Mellitus* | 39 (14.4) | 10 (13.5) | | 29 (14.7) | 0.801 |
| *BMI, median (IQL)* | 26 (24-29) | 24 (25.9-30.0) | | 24 (26.1-29) | 0.770 |
| *Active smoking* | 74 (27.3) | 18 (24.3) | | 56 (28.4) | 0.499 |
| *Stopped smoking after event* | 42 (15.5) | 8 (10.8 | | 34 (17.3) | 0.403 |
| *Alcohol use* | 156 (57.6) | 38 (52.1) | | 118 (61.1) | 0.314 |
| *Heavy drinker* | 64 (23.6) | 12 (16.4) | | 52 (27.3) | 0.067 |
| ***Type of event, N (%)*** | | | | | |
| *NSTEMI* | 155 (57.2) | 49 (66.2) | | 106 (53.8) | 0.066 |
| *STEMI* | 116 (42.8) | 25 (33.8) | | 91 (46.2) | 0.066 |
| **Cardiac intervention*, N (%)*** | | | | | |
| *PCI* | 218 (80.4) | 56 (75.7) | | 162 (82.7) | 0.195 |
| *CABG* | 31 (11.4) | 3 (4.1) | | 28 (14.4) | 0.018 |
| *None* |  |  | |  |  |
| ***Measurements, mean ±SD*** | | | | | |
| *TC, mmol/L* | 4.9 (1.16) | 5.4 (1.1) | | 4.7 (1.1) | <0.001 |
| *SBP, mmHg (median - IQR)* | 142 (127-160) | 154 (134-168) | | 137 (124-156) | <0.001 |
| *DBP, mmHg* | 86.8 (15.6) | 88.3 (1.9) | | 86.2 (1.1) | 0.335 |
| ***Previous CVD history, N (%)*** | | | | | |
| *CABG* | 4 (1.5) | 1 (1.4) | | 3 (1.5) | 0.917 |
| *PCI* | 32 (11.8) | 10 (13.5) | | 22 (11.2) | 0.594 |
| *ACS* | 37 (13.7) | 12 (16.2) | | 25 (12.7) | 0.451 |
| *Angina pectoris* | 39 (14.4) | 26 (13.2) | | 13 (17.6) | 0.361 |
| *PAD* | 14 (5.2) | 4 (5.4) | | 10 (5.1) | 0.913 |
| *Stroke* | 6 (2.2) | 3 (4.1) | | 3 (1.5) | 0.207 |
| *CKD* | 11 (4.1) | 5 (6.8) | | 6 (3.1) | 0.168 |
| ***Logistical*, median (25th and 75th percentiles)** | | | | | |
| *Distance to CR km* | 6.4 (3.3 – 10.1) | 6.1 (3.5-9.9) | | 6.4 (3.2-10.2) | 0.963 |
| *Driving distance km* | 9.5 (4.9 – 14.2) | 9.4 (5.0-14.1) | | 9.5 (4.6-14.2) | 0.805 |
| *Driving duration min* | 12 (8.1 – 16.2) | 11.9 (8.6-16.6) | | 12.1 (8.0-16.1) | 0.980 |
| ***Social economic status, N (%)*** | | | | |  |
| *Low* | 89 (32.8) | 60 (34.9) | | 29 (29.3) | 0.345 |
| *Moderate* | 90 (33.2) | 59 (34.3) | | 31 (31.3) | 0.615 |
| *High* | 92 (34.0) | 53 (30.8) | | 39 (39.4) | 0.151 |

Abbreviations: SD, standard deviation; BMI, body mass index; PCI, percutaneous coronary intervention; SBP, systolic blood pressure; DBP, diastolic blood pressure; CABG, coronary artery bypass graft surgery; TC, total cholesterol; IQR, interquartile range; ACS, acute coronary syndrome; PAD, peripheral artery disease; CKD, chronic kidney disease

* - Data missing for 30 patients.

** - Data missing for 32 patients.

**Supplementary Tab. 3** Comparison between sex and CR participation on MACE

|  | **Female** | | | **Male** | | |  |
| --- | --- | --- | --- | --- | --- | --- | --- |
| Variables, N (%) | CR participants (N= 39) | Non-CR participants (N=35) | P-value | CR participants  (N= 133) | Non-CR participants  (N= 64) | P-value | Sex interaction P-value |
| *Any MACE* | 6 (15.4) | 7 (20.0) | 0.602 | 13 (9.8) | 10 (15.6) | 0.231 | 0.775 |
| *All-cause mortality* | 0 (0.0) | 3 (8.6) | 0.062 | 2 (1.5) | 4 (6.3) | 0.069 | n.c.* |

Abbreviations: CR, cardiac rehabilitation; MACE, major adverse cardiac events.

* - P-value could not be calculated due to a zero cell count
